# Supplementary material for: Maternal Autogenous Inactivated Virus Vaccination Boosts Immunity to PRRSV in Piglets
Source: Vaccines (Basel). 2021 Jan 31;9(2):106. doi: 10.3390/vaccines9020106 (PMC7912564; doi:10.3390/vaccines9020106)
Supplement: Supplementary file 1 [file vaccines-09-00106-s001.pdf]

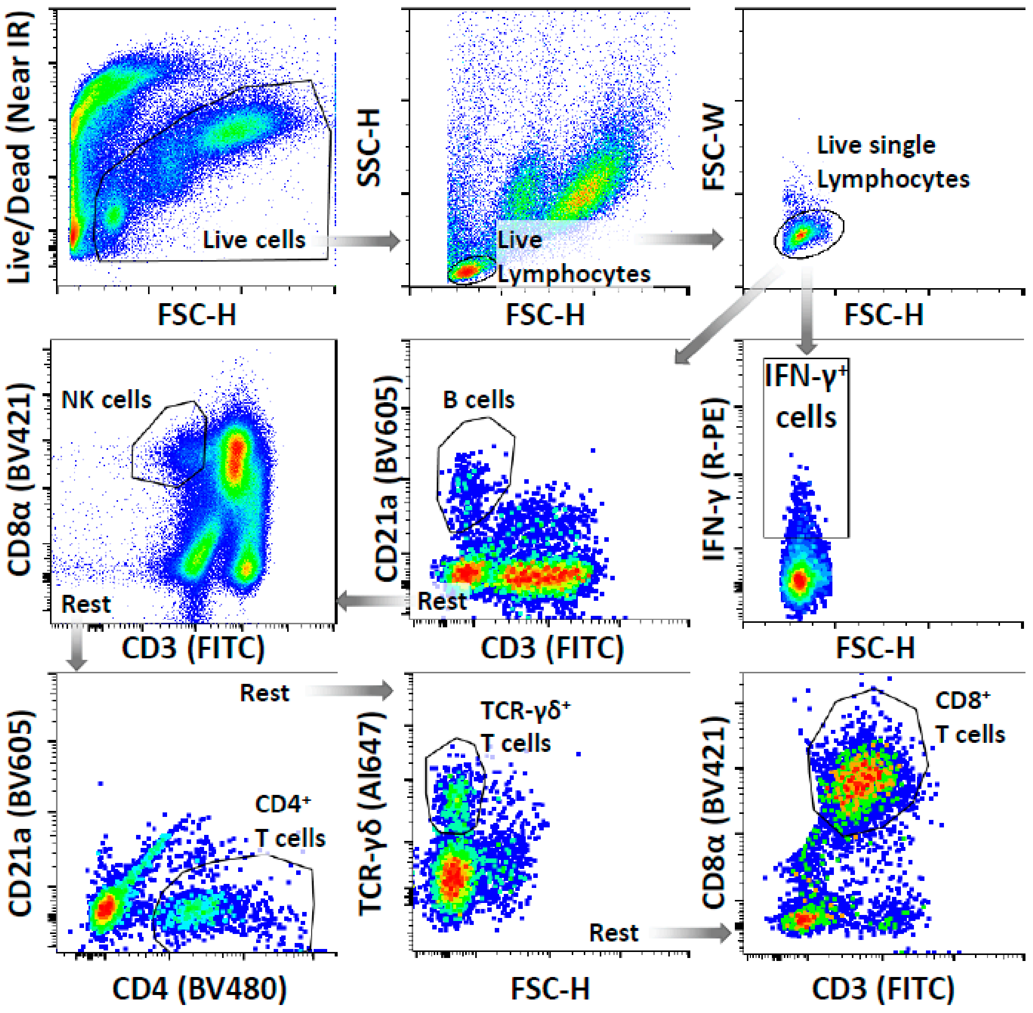

Figure S1. Gating hierarchy for IFN-γ response to PRRSV in BAL (shown), lung tissue and tracheobronchial lymph nodes: Due to a higher autofluorescence of some cells isolated from BAL, lung and lymph node tissue, the gating hierarchy had to be adapted. Dead cells were excluded by a Live/Dead discrimination dye. Next, live lymphocytes were gated based on their size (FSC-H) and granularity (SSC-H). FSC-H was FSC-W was used to exclude doublets to ensure the further analysis is performed on single living lymphocytes (SLLs). The IFN-γ expression of these SLLs was analyzed via FSC-H vs IFN-γ-R-PE. As well, SLL immune cell subsets were further discriminated into B cells (CD3-CD21a+); the non-B cells were used to gate for NK cells (CD3-CD8α+); the remaining non-B-non-NK cells were used to gate on CD4 T cells (CD4+CD21a-), the non TCR-γδ T cells (FSC-H$_{low}$TCR-γδ+) and CD8 T cells (CD3+CD4-TCR-γδ-CD8α+). These immune cell subgates were then applied to IFN-γ+ cells to determine the contribution of each subset to the overall IFN-γ production in SLLs.

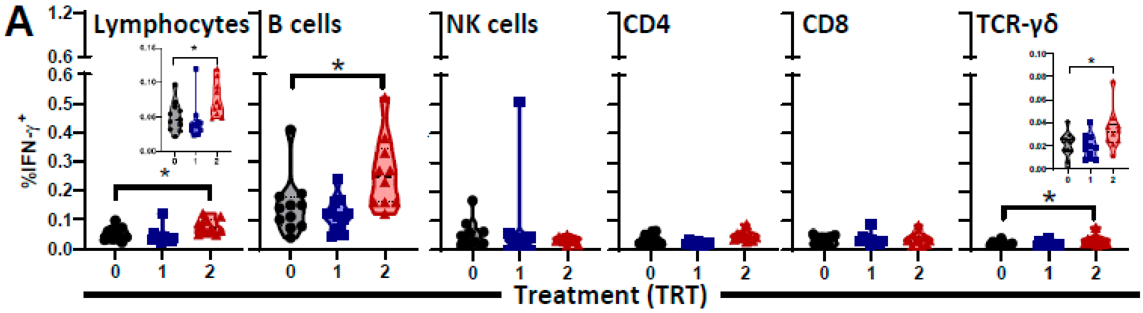

Figure S2. Systemic IFN-γ response to PRRSV1-7-4 in pre-challenges piglets at 2 weeks of age (0 wpi). The IFN-γ production of blood immune cell subsets is shown as a percentage of all SLLs, B cells, NK cells, CD4, CD8, and TCR-γδ T cells (from left to right). Data were analyzed via 1-way ANOVA with Dunnett's multiple comparisons. Significant differences between treatments are designated as * p <0.05.
